# Feeling the heat: the effect of acute temperature changes on predator–prey interactions in coral reef fish

**DOI:** 10.1093/conphys/cov011

**Published:** 2015-03-16

**Authors:** Bridie J. M. Allan, Paolo Domenici, Phillip L. Munday, Mark I. McCormick

**Affiliations:** 1 ARC Centre of Excellence for Coral Reef Studies, James Cook University, Townsville, QLD 4811, Australia; 2 College of Marine and Environmental Sciences, James Cook University, Townsville, QLD 4811, Australia; 3 CNR-IAMC, Istituto per l'Ambiente Marino Costiero, Località Sa Mardini, Torregrande (Oristano), Italy

**Keywords:** Climate change, coral reef fish, locomotory performance, predator–prey interaction

## Abstract

Recent studies demonstrate that the elevated temperatures predicted to occur by the end of the century can affect the physiological performance and behaviour of larval and juvenile fishes; however, little is known of the effect of these temperatures on ecological processes, such as predator–prey interactions. Here, we show that exposure to elevated temperatures significantly affected the predator–prey interactions of a pair of common reef fish, the planktivorous damselfish (*Pomacentrus wardi*) and the piscivorous dottyback (*Pseudochromis fuscus*). When predators exposed to elevated temperatures interacted with prey exposed in a similar manner, maximal attack speeds increased. This effect coupled with decreasing prey escape speeds and escape distances led to increased predation rates. Prey exposed to elevated temperatures also had decreased reaction distances and increased apparent looming threshold, suggesting that their sensory performance was affected. This occurred despite the increase in maximal attack speeds, which in other species has been shown to increase reaction distances. These results suggest that the escape performance of prey is sensitive to short-term increases in ambient temperature. As marine environments become more thermally variable in the future, our results demonstrate that some predators may become more successful, suggesting that there will be strong selection for the maintenance of maximal escape performance in prey. In the present era of rapid climate change, understanding how changes to individual performance influence the relationships between predators and their prey will be increasingly important in predicting the effects of climate change within ecosystems.

## Abstract (precis)

Exposure to elevated temperatures significantly affects the predator-prey interactions of a common pair of reef fish. Predators exposed to elevated temperatures had increased maximum attack speeds. This coupled with decreasing prey escape speeds and distances led to increased predation rates with subsequent increases in capture success.

## Introduction

Climate change models predict that sea surface temperatures are set to increase by up to 3°C by the end of the century owing to rising atmospheric greenhouse gases (Collins *et al.*, 2013). However, most marine organisms already experience temperature variation as a result of thermal fluctuations at a range of temporal and spatial scales resulting from changing seasons, currents, upwelling, tides, local topography and daily changes in solar radiation (Soon and Legates, 2013). For coral reefs, which are considered to be thermally stable environments, temperatures also change both temporally and spatially. These changes are driven by the shallow, sloping and rough bottom topography (Monismith *et al.*, 2006) typical of coral reefs that alter flow regimes, as well as predictable changes in solar radiation (Leichter *et al.*, 2006) and cloud cover (Leahy *et al.*, 2013). Thermal regimes on coral reefs have been observed to change by up to 4–8°C on a diurnal cycle in some places, with peaks of up to 12°C during the summer months (McCabe *et al.*, 2010), and by up to 9°C on a seasonal basis at higher latitudes (Rummer *et al.*, 2013), exceeding those temperatures that are predicted under current climate change scenarios (Collins *et al.*, 2013). Assessing the effects of natural thermal variability now can be important for predicting future impacts of climate change on marine organisms.

Ambient temperatures strongly influence a broad range of physiological and behaviourial traits in all marine organisms. For most marine organisms, temperature is a major environmental determinant of life-history processes and governs basic physiological functions, such as swimming performance (Rome, 2007), growth (Pauly, 1980), neural control (Szabo *et al.*, 2008) and behaviour (Biro *et al.*, 2010). Fluctuating temperatures can have an effect on swimming performance through changes in aerobic scope (Farrell, 2002; Johansen and Jones, 2011), cardiac output (Eliason *et al.*, 2011), muscle development (Hanel and Wieser, 1996) and the contractile properties of the swimming muscles (Wakeling, 2006).

The mechanisms underlying these changes are complex and arise from changes in the balance between ATP consumption and generation as well as direct effects on ligand binding, diffusion and enzyme catalysis (Cossins and Bowler, 1987). Elevated temperatures lead to an increase in metabolic rate, with subsequent changes in energy requirements (Clarke and Johnston, 1999). This can result in reduced net food conversion, ultimately affecting muscle development (Johnston *et al.*, 2001) through trade-offs between hypertrophy (an increase of mass of individual fibres) and hyperplasia (an increase in the number of fibres; Hanel and Wieser, 1996). Furthermore, neural control of antipredator swimming behaviour was found to be affected by temperature (Szabo *et al.*, 2008).

Thermal fluctuations cause changes not only in physiological processes but also in the behaviourial capacities that are directly linked to them, such as activity (Biro *et al.*, 2010), sensory responsiveness (Webb and Zhang, 1994) and the interactions between predators and their prey (Freitas *et al.*, 2007; Grigaltchik *et al.*, 2012). A critical stage in the life of reef fishes is at the end of the larval phase, when they settle to the benthic environment. Mortality schedules during the first few days of settlement are high, averaging 56% for tropical reef fishes (Almany and Webster, 2006). Success at this life stage is predominantly a consequence of the size, growth and the fast-start performance of new recruits (Green and McCormick, 2005; Holmes and McCormick, 2010; Allan *et al.*, 2013). Fast starts are short, high-energy swimming bursts and are driven by the rapid contraction of the white muscle fibres (Rome *et al.*, 1988; Domenici and Blake, 1997). White muscle fibres contain fewer mitochondria than red muscle; therefore, they rely largely on anaerobic metabolism to power them (Josephson, 1993). The fast kinematics of escape responses are usually controlled by the large Mauthner neurons, which are triggered as a reaction to the fast approach of a predator, although other reticulospinal cells may also be involved (Eaton *et al.*, 2001). Successful fast starts consist of finely tuned responsiveness and locomotor performance (Domenici, 2010). However, fast starts are sensitive to changes in ambient temperatures, with responses differing between species and ontogenetic stages (for review see Johnston and Temple, 2002; Wilson, 2010), as well as between predators and their prey due to changes in their temperature tolerance and sensitivity (Freitas *et al.*, 2007; Grigaltchik *et al.*, 2012).

Acute heat stress, defined as physiological stress associated with short-term changes in ambient temperature, can lead to an increase in prey vulnerability (Yocom and Edsall, 1974) as well as an increase in attack rates from predatory fish (Persson, 1986). However, it is unknown whether the modest temperature increases that an animal may naturally experience within its environment will influence predator–prey interactions in coral reef fishes. As a result of the relative effects of elevated temperature exposure on predators and prey, there may be changes in predator strike success or prey escape rates. Consequently, this could lead to changes in predation pressure, which could translate into changes in community structure and function. The aim of the present study, therefore, was to determine whether predators and prey respond differently to modest increases in temperature, and whether this changes the outcome of predator–prey interactions by affecting the kinematics of their responses to one another.

## Materials and methods

### Study site, fish collection and maintenance

Fishes were collected during October 2012 at Lizard Island (14° 40′ S, 145° 28′ E) in the northern Great Barrier Reef, Australia. Temperature loggers (Sensus ultra) deployed around Lizard Island for the 3 years before the study found that water temperatures in the shallow (<10 m) water where fish were collected from ranged from 20.6 to 30.6°C and had a diurnal range of 1.2°C during the summer months, when fish recruitment occurs. During the recruitment period of 2012, the water temperature ranged from 25.2 to 29.2°C. The Ward's damselfish, *Pomacentrus wardi* (Pomacentridae), was used as the prey species and is a small planktivorous fish commonly found on Indo-Pacific coral reefs. The dottyback, *Pseudochromis fuscus* (Pseudochromidae), was used as the predator. *Pseudochromis fuscus* is widely distributed throughout the Indo-Pacific and is an important predator of newly settled coral reef fishes (Feeney *et al.*, 2012), including *P. wardi*. Newly metamorphosed *P. wardi* [range 11.2–15.4 mm, 13.6 ± 1.3 mm mean standard length (SL) ± SD] were collected using light traps moored 100 m off the fringing reef of Lizard Island. On the morning of capture, *P. wardi* individuals were transferred into tanks at ambient, control temperatures (26.7°C). These individuals were then split into control (present-day temperature, 26.7°C ± 0.1°C) and treatment groups (elevated temperature, 29.6°C ± 0.1°C). For fish in the +3°C temperature group, the temperature was raised by 1°C every 8 h until the final temperature of ∼29.6°C was reached to avoid any stress associated with rapid temperature increases. Fish were maintained in these treatments for a period of 7 days, because we were interested in behavioural changes associated with short-term increases in temperature. A week is sufficient for thermal acclimation to occur in reef fishes, and previous studies have not found significant improvement of physiological processes after longer exposures to elevated temperatures (Nilsson *et al.*, 2010). Tanks were heated with 300 W bar heaters and insulated to ensure stability of the chosen temperatures of 26.7 and 29.6°C. Fish were fed four times daily *ad libitum* with newly hatched *Artemia* sp. but were starved for the 12 h prior to commencement of experimental trials to standardize for satiation. A 12 h light–12 h dark regimen was used.

Adult *P. fuscus* (range 63–96 mm, 76.9 ± 9.7 mm mean SL ± SD) were collected with a dilute solution of clove oil (Munday and Wilson, 1997) from around the shallow fringing reef off Lizard Island. Immediately after collection, fish were transported back to the Lizard Island Research Station, where they were housed separately in mesh breeding baskets within 30 litre aquaria to avoid aggressive interactions. Fish were maintained in tanks for 7 days (following the same protocol as *P. wardi*) and were fed two juvenile reef fish morning and night and then not fed for the last 24 h prior to the interaction trial to standardize for satiation.

### Interaction trials

Experimental trials were conducted over a period of 10 days in a temperature-controlled room at the Lizard Island Research Station. Trials were conducted at the same water temperature as the acclimation temperature for the test fish. Predator–prey interactions were measured using a standard protocol established by Allan *et al.* (2013). Briefly, this involved placing a predator and a prey fish into an experimental arena (38 cm × 58 cm × 10 cm water height) and filming the ensuing interaction at high speed (420 frames s^−1^) for 10 min or until the prey had been consumed. Trials commenced only when the predator was at the opposite end of the tank to the prey at the start of the interaction to standardize for predator position. Kinematic variables were measured based on the centre of mass (COM) of the fish when stretched straight, based on Webb (1976). The COM was assumed to be at 35% of the body length from the tip of the snout as it is the case for generalist fish (Domenici and Blake, 1997). Stages 1 and 2 were defined by directional changes of the anterior part of the body of the fish, based on Domenici and Blake (1997). Predator attacks were measured only when a predator showed a fast-directed burst towards the prey (>3 body lengths s^−1^). All variables, with the exception of number of prey caught, were measured using only the first attack that occurred within a trial. This was done to control for any anaerobic stress either the predator or prey may have experienced due to prolonged attacks. Both predators and prey were used once to avoid habituation to the experimental procedure. Prey suffering was minimal because prey were consumed immediately following a successful strike.

The following performance variables were measured.

#### Prey

- Prey reaction distance (RD; in metres), i.e. the distance between the prey COM and the tip of the predator's snout at the onset of the escape response to a predator attack.
- Apparent looming threshold (ALT) was defined as the apparent looming threshold for prey avoidance responses to a predatory strike and is a measure of the magnitude of the prey's response to the perceived threat of predation. The higher the perceived threat, the higher the ALT (in radians per second) measured at the onset of the escape response and measured as the rate of change of the angle (α) subtended by the predator's frontal profile as seen by the prey. Previous work has shown that fish tend to react to an approaching stimulus (a predator) when a given threshold of dα/d*t* (i.e. ALT) is reached. The ALT is calculated as (4*US*)/(4*D*^2^ + *S*^2^), based on (Dill, 1974) and (Webb, 1982); where *U* is the predator speed, calculated as the speed of the predator in the frame prior to the prey's response; *S* is calculated based on the morphological characteristics of the predator, i.e. *S* = (maximal depth + maximal width)/2, whereby both maximal depth and maximal width are at 0.25 lengths of the predator (personal observation); and *D* is  the reaction distance calculated between the prey COM and the point on the predator where its maximal width is located. Hence, *D* = RD + 0.25 lengths of the predator. As a consequence, for any given predator speed and morphology, as RD decreases ALT increases.
- Prey escape distance (in metres), i.e. the straight-line distance between the prey COM at the onset of the escape response and at the end of the escape response (i.e. when the prey came to a halt).
- Maximal prey escape speed (in metres per second), i.e. the top speed achieved at any point in time during the escape response, measured using the prey COM.
- Mean prey escape speed (in metres per second) was measured as the distance covered within a fixed time (24 ms). This fixed duration was based on the average duration (22.8 ms) of the first two flips of the tail (the first two axial bends, i.e. stages 1 and 2 defined based on Domenici and Blake, 1997), which is the period considered crucial for avoiding ambush predator attacks (Webb, 1976).

#### Predator

(vi) Capture success, i.e. the percentage of trials in which the predator ingested the prey within the 10 min filming period, out of the total number of trials for each treatment.
(vii) Predation rate, i.e. capture success divided by the number of attacks per unit time.
(viii) Attack rate, i.e. number of attacks per unit time, measured for each interaction.
(ix) Predator attack distance (in metres), i.e. the straight-line distance between the predator COM at the time the attack commenced and the end of the attack (end is defined as when the predator came to a halt).
(x) Maximal predator attack speed (in metres per second), i.e. the top speed achieved at any point in time during the attack, based on the predator COM.

### Statistical analyses

The effects of elevated temperatures on performance kinematics were tested separately for prey and predators using one-factor MANOVAs. One-way ANOVAs were performed to determine the nature of any differences found by the MANOVA. Residual analysis indicated that data met the assumptions of normality and homogeneity of variance. To test the null hypothesis that predator capture success is independent of predator and prey temperature exposure, capture success was compared by 2 × 2 contingency table analysis. Predation rate data did not meet the assumption of homogeneity of variance; therefore, a Kruskal–Wallis test was performed to explore differences in predation rates between the two treatments.

## Results

### Prey

**Figure 1:**
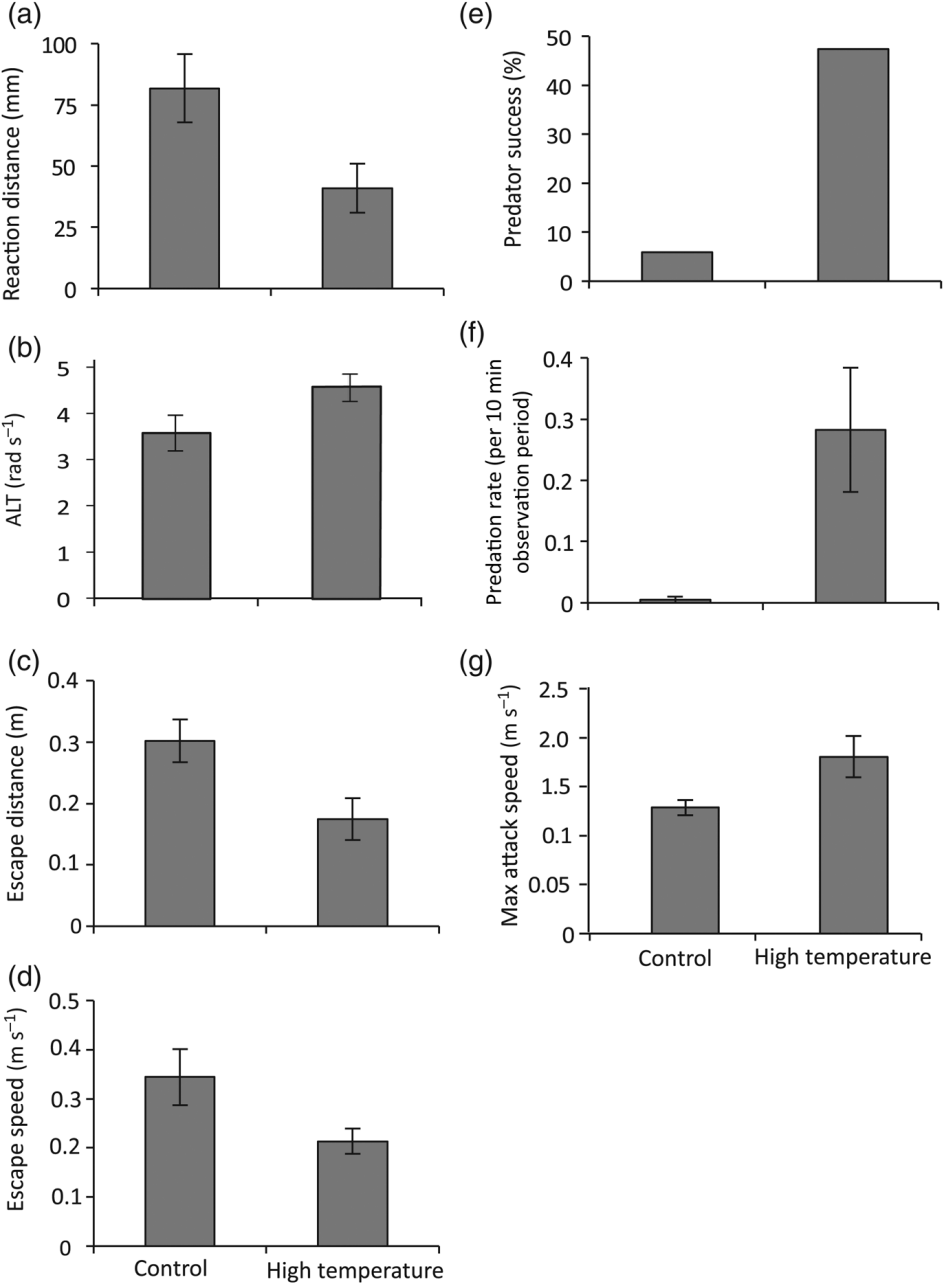
Comparison of the effects of temperature (26.7 and 29.6°C) on interactions between a predator (*Pseudochromis fuscus*) and prey (*Pomacentrus wardi*), as follows: prey reaction distance (**a**); prey apparent looming threshold (ALT; **b**); prey escape distance (**c**); prey speed (**d**); predator success (**e**); predation rate (**f**); and maximal attack speed (**g**). *n* = 17 for control temperature and *n* = 19 for high temperature. Error bars are SEM.

The MANOVA revealed a significant effect of temperature on prey escape performance (Pillai's trace_4,31_ = 5.49, *P* = 0.001). One-factor ANOVAs detected significant differences in four out of the five tested behaviourial attributes, namely RD, ALT, escape distance and mean prey escape speed. The RD of the prey to the predator at the onset of the first attack was affected by exposure to elevated temperatures (Fig. 1a; *F*_1,34_ = 5.40, *P* = 0.02). Specifically, prey exposed to elevated temperatures allowed exposed predators in a similar manner to get twice as close to them before undertaking an escape response (82 compared with 40 mm). The ALT was also significantly higher for prey following exposure to elevated temperatures (Fig. 1b; *F*_1,34_ = 7.30, *P* = 0.01). The distance travelled during an escape response (escape distance) was significantly shorter than when interactions occurred at the control temperatures, demonstrating an acute effect of elevated temperature exposure (Fig. 1c; *F*_1,34_ = 6.87, *P* = 0.01). Exposure to elevated temperatures also significantly affected the mean response speed of the prey, with prey exposed to elevated temperatures being considerably slower in comparison to the control temperature group (Fig. 1d; *F*_1,34_ = 5.35, *P* = 0.02). There was no significant difference in the maximal speed achieved between the two treatment groups.

### Predator

Capture success was significantly associated with the elevated temperature treatment experienced by the predator and the prey (Fig. 1e; χ^2^ = 8.95, d.f. = 3, *P* = 0.005). Within this treatment, capture success was 47%; 41% greater than the pairs exposed to the control temperature (5.8% capture success). Exposure to elevated temperatures also significantly affected predation rates (Fig. 1f; *H* = 7.81, d.f.= 1, *P* = 0.005), with a markedly higher predation rate in the predators exposed to the elevated temperature compared with the control temperature. A MANOVA revealed a significant effect of temperature on the attack characteristics of the predators (i.e. attack rate, attack distance and maximal attack speed; Pillai's trace_3,32_ = 5.04, *P* = 0.002). ANOVAs detected significant differences only in maximal speed achieved (Fig. 1g; *F*_1,34_ = 4.89, *P* = 0.03), with predators exposed to elevated temperatures reaching greater speeds than those exposed to the control temperature. Exposure to elevated temperatures did not significantly influence the distance travelled during an attack or the attack rate.

## Discussion

Fish experience temperature fluctuations at a range of temporal and spatial scales that influence their physiology (Rome, 2007) and behaviour (Biro *et al.*, 2010). Our results demonstrate that even modest temperature increases can lead to changes in the timing, kinematics and outcome of predator–prey interactions. Specifically, prey exposed to elevated temperatures for 7 days exhibited changes in locomotor performance, with decreases in swimming speeds and reduced escape distances. This reduced prey performance is likely to be due to a decline in muscle power at elevated temperatures (Johnson and Bennett, 1995). Many species exhibit a decline in swimming performance once a thermal threshold has been reached (for review see Johnston and Temple, 2002), with species responses differing due to the thermal sensitivity of their performance curves (Grigaltchik *et al.*, 2012).

Alternatively, the mechanistic explanation underlying the observed responses may be declining aerobic scope caused by an increase in resting metabolic rate due to exposure to elevated temperatures (Munday *et al.*, 2012). Although fast starts are powered anaerobically, this energy debt has to be paid off by post-exercise oxygen consumption, which requires more energy than used initially, creating an energy deficit (Moyes *et al.*, 1993). Fishes with reduced aerobic scope in elevated temperature conditions may therefore show low-performance escapes as an energy-saving strategy (Johansen and Jones, 2011). Moreover, reduced aerobic scope may have caused a decrease in burst-swimming performance indirectly, i.e. as a result of reduced body condition.

In addition to reduced locomotor performance, there were changes in the responsiveness of the prey, with reduced reaction distances and an increase in apparent looming threshold. The fast kinematics of escape responses are likely to be under the control of Mauthner cells, which are triggered as a reaction to the fast approach of a predator (Eaton *et al.*, 2001). Given that elevated temperatures led to decreased reaction distances and an increase in apparent looming threshold, the motivational component of the motor response, which also acts upon the duration of the burst, may have been affected. Furthermore, it is also possible that the elevated temperature had a negative effect on the neural control and therefore the timing of the response. Webb and Zhang (1994) found that the responsiveness (i.e. the reaction distance) of prey (goldfish; *Carassius auratus*) to an attacking predator (rainbow trout; *Oncorhynchus mykiss*) was affected following acute exposure to elevated temperatures, leading to increased predator success. Szabo *et al.* (2008) found that acclimation to high temperatures altered the escape responses of *C. auratus* through changes in the cellular physiology of the Mauthner cell circuit, which led to differences in the balance between the excitatory and inhibitory transmission onto the Mauthner cell. Fish acclimated to warmer water tended to show high excitability but a lower directional discrimination of the stimulation. These studies indicate that sensory responsiveness, which is a crucial factor for successfully escaping from predators (Domenici, 2010), can be affected by temperature changes.

Predators exposed to the elevated temperature had an increase in capture success compared with those at the control temperature, which may be due to two factors. Firstly, the reactivity and locomotor performance associated with the escape behaviour of the prey decreased following exposure to the elevated temperature and secondly, there was an increase in the predation rate and maximal attack speeds, which could be interpreted as an increase in predator motivation to capture prey. Hunger may have played a role in determining motivation, because the energetic demands of the predator may have increased due to an increase in metabolic rate, while food availability was kept constant in the two treatment groups. In addition, Webb (1984) showed that some predators attack at submaximal speeds; therefore, it is possible that in the present study increased hunger (resulting from the temperature-induced increase in metabolic rate) may have caused predators to attack at a higher speed compared with the control predators, because they may not have exhibited maximal attack speeds in the control treatment. Furthermore, Webb (1984) and Domenici and Blake (1997) suggest that the strategy of striking at speeds below maximum ensures that prey do not initiate an escape response early (i.e. with a short reaction distance), which could result in displacing prey further from the predator's strike trajectory. Interestingly, we found that despite the increase in predator attack speed (which usually causes an increased prey reaction distance; Dill, 1974), prey exposed to the elevated temperature showed a reduction in reaction distance, as a result of their decreased sensory performance (i.e. increase in ALT).

Although it is possible that predators increased their attack speeds at high temperature as a result of both increased hunger level and submaximal speeds in the control situation, additional physiological considerations may also explain the differential thermal response of predator and prey. Given that only one of the locomotory variables we tested for our predator (maximal attack speed) was affected following exposure to the elevated temperature, this suggests that the attack performance of *P. fuscus* was robust to modest temperature increases. This may be because they have experienced a wider breadth of temperatures on the shallow fringing reef than the prey, which were returning to the reef from the open ocean at the end of their larval phase. Alternatively, there may have been differential levels of thermal acclimation between the two species as a product of their recent and/or evolutionary history. Both predators and prey were exposed to the elevated temperature for a minimum of 7 days before predation trials commenced, which should have allowed acclimation to occur, but the capacity for thermal acclimation may differ between the two species. However, if fishes are exposed to temperatures for longer than 7 days, then the potential for acclimation to occur may increase (Johnson and Bennett, 1995). Acclimation can lead to changes in the speed of contraction of fast muscle fibres and a corresponding change in swimming performance (Rome, 1985; Heap and Goldspink, 1986; Johnson and Bennett, 1995). Fishes that can acclimate to increasing temperatures usually experience increased capture or escape success (Yocom and Edsall, 1974; Beddow *et al.*, 1995).

Overall, we found an increase in predation rate in response to a temperature increase that was within the range naturally found at the study site (ranging from 25.2 to 29.2°C during the 2012 recruitment period). This change in predator success resulted from a negative effect of temperature on the fast-start kinematics and responsiveness of the prey, while the locomotor performance of the predator was enhanced. As a consequence of elevated temperatures affecting the prey and predator in opposite ways, predator strike success increased at elevated temperatures. Regardless of the possible mechanistic explanations for the changes we saw, the differing responses between *P. wardi* and its predator *P. fuscus* to small temperature increases could have consequences for ecological interactions and the relative abundance of species within coral reef fish communities. How critical these changes are, particularly when combined with other stressors, remains to be determined. In an era of rapid climate change, understanding small-scale changes in ambient temperatures and how these influence the interaction between organisms and their environment will be increasingly important in predicting the effects of climate change within ecosystems. We have demonstrated that the kinematics at the basis of predator–prey interactions in coral reef fish are significantly changed following exposure to a modest increase in temperature within the range normally experienced. Further studies should test how predator–prey interactions change at temperatures beyond their current limits to determine the extent to which the physiological mechanisms that underpin performance will affect key ecological processes in the future.

## Funding

This work was supported by the Australian Research Council Centre of Excellence for Coral Reef Studies and an Australian Research Council Discovery grant (DP120101993).
